# Induction of cell apoptosis by biliverdin reductase inhibitor in MCF-7 and MDA-MB-468 breast cancer cell lines: Experimental and in silico studies

**DOI:** 10.17179/excli2021-4069

**Published:** 2021-10-21

**Authors:** Seyedeh Zahra Shahrokhi, Fatemeh Soghra Karami Tehrani, Siamak Salami

**Affiliations:** 1 Department of Clinical Biochemistry, Faculty of Medical Sciences, Tarbiat Modares University, Tehran, Iran; 2 Department of Clinical Biochemistry, School of Medicine, Shahid Beheshti University of Medical Sciences, Tehran, Iran

**Keywords:** biliverdin reductase-A, breast cancer cell lines, caspase activity, molecular docking, high-performance liquid chromatography

## Abstract

Biliverdin reductase, biliverdin and bilirubin are known as important components of cellular signaling pathways that play major roles in cell proliferation and apoptosis, although their physiological relevance is still under evaluation. This study was designed to investigate the expression and activity of BVR-A and its apoptotic effect in the breast cancer cell lines, MCF‐7 and MDA‐MB‐468. The expression of BVR-A was examined by real‐time PCR and western blot analysis. Bilirubin concentration was measured by HPLC and molecular docking was performed to identify an appropriate inhibitor for BVR-A. To detect cell apoptosis, annexin V‐PI staining, caspase-3, -8, and -9 activities were evaluated. Cell viability was reduced by biliverdin, in a dose‐dependent manner, and an intrinsic apoptotic response occurred which was evidenced by caspase‐3 and -9 activities. The intra- and extracellular concentrations of bilirubin were higher in MCF-7 cells than those of MDA-MB-468 cells. The expression of BVR-A, at mRNA and protein levels, in MCF-7 was also higher than that of MDA-MB-468 cells. Treatment of both cell lines with biliverdin plus DTNB, a BVR-A inhibitor, increased the cell death significantly when compared with biliverdin alone. Using annexin V-PI staining and assessment of caspase‐3 activity, it was confirmed that biliverdin together with DTNB increases apoptosis in breast cancer cells. In conclusion, biliverdin has an important role in cell apoptosis and inhibition of biliverdin reductase increases the apoptotic effect of biliverdin.

## Introduction

In the metabolic pathway of heme, biliverdin is converted to bilirubin by biliverdin reductase (BVR) (Gazzin et al., 2016[8]). Recent data indicates that BVR, biliverdin, and bilirubin are important in the cellular signaling pathways such as mitogen-activated protein kinase (MAPK) pathway (Brito et al., 2008[4]; Florczyk et al., 2008[7]). BVR-A, the major isoform of BVR, is a multifunctional enzyme that possesses biological functions such as cell growth and apoptosis and, therefore, it might be involved in the pathogenesis of cancers (Kim et al., 2011[14]; Zhang et al., 2018[33]). Concerning BVR-A expression in malignant cells, little is known, although its expression has been reported in lung and breast cancer cells (Zhang et al., 2018[33]). It has been shown that the expression of BVR-A is cell-specific and is important in cell growth and apoptosis (Wada and Penninger, 2004[29]; Barone et al., 2011[2]) since its over-expression has been found in the arrested cells in G1/G0 phase (Gibbs and Maines 2007[9]). However, there is evidence that BVR-A could prevent cell apoptosis by suppression of death receptor-5, cytochrome c, and caspase-3 activity (Pachori et al., 2007[21]).

Recent studies have shown that biliverdin modulates cellular growth in hyper-proliferative diseases such as cancers (Ollinger et al., 2007[19]; Zheng et al., 2014[34]). It regulates the activity of several enzymes such as mitogen-activated protein kinase (MAPK) that is involved in signal transduction pathways in both normal and malignant cells (Gibbs et al., 2012[10]; Loboda et al., 2015[16]). The anti-proliferative effects of biliverdin have also been shown by inhibition of MAPK phosphorylation and arresting the cell cycle at G0/G1 phase (Zheng et al., 2014[34]). Furthermore, biliverdin suppresses the expression of cyclin A, D1, and E and also reduces phosphorylation of retinoblastoma (Rb), a tumor suppressor protein (Ollinger et al., 2005[18]). Interestingly, it has been demonstrated that biliverdin has anti-apoptotic effects in some tumor cells (Busserolles et al., 2006[6]; Parfenova et al., 2006[22]; Bulmer et al., 2008[5]). In serum-deprived Caco-2 cells, biliverdin significantly inhibited cell apoptosis (Busserolles et al. 2006[6],) and pretreatment of renal cell by biliverdin increased cell resistance to the induction of apoptosis by cisplatin through inhibition of oxidative stress (Lv et al., 2016[17]).

Biliverdin was shown to possess highly potent antioxidant properties (Zheng et al., 2014[34]). Most effects of biliverdin are mimicked by bilirubin, although the signaling pathways such as the effects on BVR-A are mostly different (Wegiel and Otterbein, 2012[30]). Interestingly, biliverdin is an endogenous activator of aryl hydrocarbon receptor (AhR) which contributes to its anticancer effects (Gazzin et al., 2016[8]). AhR has a potential role in the regulation of cell proliferation, cell-cycle distribution, and cell apoptosis (Puga et al., 2009[24]) and may mediate mechanisms that contribute to anticancer effects of biliverdin as reported in recent studies (Phelan et al., 1998[23]; Yin et al., 2016[31]) 

The purpose of this investigation is to examine the effects of BVR-A inhibition and biliverdin on cell growth or apoptosis in human breast cancer cell lines: MCF-7 and MDA-MB-468. Since an appropriate BVR-A inhibitor is not commercially available (Jansen et al., 2010[11]), therefore, molecular docking was applied to identify an appropriate inhibitor for BVR-A.

## Materials and Methods

### Molecular docking

In the present study, the crystal structure of human BVR-A, in complex with NADPH (PDB code: 2H63), was downloaded. The BVR-A, as a receptor molecule, was prepared for the molecular docking process by removing water, adding polar hydrogen bonds, and charge distribution. The binding site of BVR-A was detected by Autodock software. Twenty compounds reported in previous studies were selected (Zheng et al., 2014[34]; Van Dijk et al., 2017[28]) and their structures were obtained from the ZINC database (http://zinc.docking.org/) and PubChem (https://pubchem.ncbi.nlm.nih.gov). In order to rank the best ligand, in terms of orientation and binding to the active site of BVR-A, all compounds were sorted according to the pharmacophore model, using the Molecular Operating Environment software package (MOE). 

The docking process was performed with a flexible ligand and a rigid receptor. The best protein-ligand complex with lower binding energy was selected and subsequently used in Molecular dynamics (MD) simulation. MD simulation was used to inhibit BVR-A with the final inhibitor, DTNB, obtained from the docking process. It was performed with Gromacs 2019 software and Gromos54a7 force field. Receptor preparation for MD simulation was performed by removing water and NADH, the addition of polar hydrogen bonds, and charge distribution. Ligand preparation was also performed using the ATB site (https://atb.uq.edu.au).

## Materials

Dulbecco's modified Eagle's medium (DMEM) and supplements (Trypsin/EDTA, fetal bovine serum (FBS), phosphate-buffered saline (PBS), and penicillin-streptomycin) were purchased from Gibco (Grand Island, NY, USA). Unconjugated bilirubin (purity ≥ 98), biliverdin hydrochloride, bovine serum albumin (BSA), tetrazolium salts (methylthiazole tetrazolium, MTT), dimethyl sulfoxide (DMSO), chloroform, and methanol (HPLC grade) were obtained from Sigma-Aldrich (St. Louis, MO, USA). Mouse anti-actin was obtained from Santa Cruz (CA, USA), rabbit anti-BVR-A from Proteintech (Manchester, UK), and horseradish peroxidase (HRP)-conjugated anti-rabbit from Bio-Rad (Hercules, CA, USA). Polyvinylidene fluoride transfer membrane (PVDF) and Enhanced chemiluminescence (ECL) reagents were purchased from Bio-Rad (Hercules, CA, USA). Caspase-3 and -8, -9 Colorimetric Assay Kits were bought from Sigma-Aldrich. MCF-7 and MDA-MB-468 breast cancer cell lines were provided by the National Cell Bank of Iran (NCBI).

### Cell culture procedure

The cells were cultured in DMEM medium supplemented with 10 % FBS, 100 U/ml penicillin, and 100 μg/ml streptomycin. They were incubated at 37 °C in a humidified incubator with 5 % CO_2_ and were harvested upon 70 % confluence using trypsin-EDTA (0.25 %). 

### Cell proliferation assay

Cell viability was evaluated using MTT (3-(4, 5-Dimethyl-2-yl)-2, 5 diphenyltetrazolium bromide) assay. They were seeded at a density of 5-6 × 10^3^ cells/well in 96-well plates. The next day, they were incubated with biliverdin for 24 or 48 hrs. Cells incubated with DMSO were used as a control. After incubation, the culture media was removed and 20 μl of MTT was added to each well and incubated at 37 °C for 3 hrs. Then, MTT media was removed, and to dissolve MTT formazan crystals, DMSO (0.2 mL) was added. The plates were incubated, in the dark, for 10 min, and the absorbance was measured at 570 nm using a microplate reader (Tecan, Austria). IC_50_ values were calculated using GraphPad statistical software 6 (CA, USA). In order to evaluate the cytotoxic effect of DTNB, cells were incubated with this compound for 12 hrs, and the MTT assay was then applied.

### Assessment of cell death 

Annexin V/propidium iodide (PI) staining assay was applied to quantify the cell death modality. 2×10^5^ cells were plated and treated with biliverdin, biliverdin plus BVR-A inhibitor, and BVR-A inhibitor. Following treatment, cells were washed twice with PBS, mixed with 500 μl of binding buffer, stained with 5 μl of annexin V and 5 μl of PI (PI 50 μg/ml) for 10 min at room temperature in the dark and then were analyzed by a FACS Calibur flow cytometer (BD Biosciences, San Jose, CA, USA). This assay distinguishes living cells from early and late apoptotic cells.

### Determination of caspase-3, -8, and -9 activities

Caspase activities were determined using colorimetric assay kits, according to the manufacturer's instructions (Sigma-Aldrich). Following treatment of the cells, they were washed with PBS and centrifuged at 600 g for 5 min. Cells were lysed in 50 µl lysis buffer on ice for 20 min, and then centrifuged at 20,000 × g for 15 min at 4 °C. Supernatants were collected and were incubated with Ac-DEVD-pNA (substrate for caspase-3), Ac-IETD-pNA (substrate for caspase-8), and Ac-LEHD-pNA (substrate for caspase-9) in a reaction buffer at 37 °C for 2 hrs. Absorbance was read at 405 nm using a microplate reader (Tecan, Austria). Protein concentration was measured using the Bradford method.

### Quantification of intra- and extracellular bilirubin 

Bilirubin, 1 mg, was dissolved in 1 mL of DMSO to achieve a stock standard solution with a concentration of 1 mg/ml. The stock standard solution of bilirubin was diluted with mobile phase to prepare working standard solutions at concentrations of 0.002, 0.005, 0.01, 0.015, 0.02, and 0.025 mg/ml. The standard calibration curve was prepared using working standards.

MCF-7 and MDA-MB-468 cells at 80 % confluence were treated with biliverdin or DMSO for 24 hrs. The cells were washed with 0.9 % NaCl and centrifuged at 250 g, 4 °C for 5 min. NaCl was removed after which the cells were quenched by 200 µl methanol followed by adding 200 µl H_2_O on ice and subsequently mixed with chloroform at -20 °C. To disrupt the cells, the pellet was frozen, allowed to thaw, and was centrifuged at 16,100×g, 4 °C for 5 min. The lower phase was then transferred to another tube (Sapcariu et al., 2014[27]). Bilirubin concentration was measured using HPLC (high-performance liquid chromatography), equipped with a diode array detector. 10 μl of the extract was run on Symmetry C18 HPLC column (3.5 µm, 4.6 mm X 75 mm, Waters, Milford, MA, USA). The mobile phase consists of two solvent mixtures, including (a) 40 % MeOH/ 60 % ammonium acetate (pH 4.5), and (b) 100 % MeOH, in a run time of 20 minutes. The gradient program was 100 % solvent A for 15 min, 100 % solvent B for 2 min, and 100 % solvent A for 3 min, with a flow rate of 1.0 mL/min. Detection of bilirubin was performed at 450 nm. For quantification of bilirubin, a calibration curve was used (Van Dijk et al., 2017[28]).

### BVR-A assay

The activity of BVR-A was determined by measuring the rate of bilirubin formation, following the manufacturer's protocol (Sigma, St. Louis, USA). An equal amount of protein (150 µg) was incubated with assay buffer at 37 °C for 5 min. Then, working solutions were added and the plate was read by Cytation™3 Cell Imaging Multi-Mode Reader (BioTek Instruments Inc, Winooski, VT, USA). The linear portion of the curve was selected for the calculation of BVR-A activity.

### RNA Extraction and cDNA synthesis

Total RNA was extracted from MCF-7 and MDA-MB-468 cell lines by RiboEx reagent (Gene All, South Korea). RNA quality and purity were determined by the nanodrop spectrophotometer (NanoDrop Technologies Inc., Wilmington, DE). Total RNA (1 µg) was reverse transcribed to cDNA by PrimeScript™ RT reagent Kit (Takara, Japan). Real-time PCR was conducted by a Step- OnePlus Real-Time PCR system (Applied Biosystems) using SYBR Green Master Mix (Takara, Japan). All assays were conducted in duplicate at 95 °C for 5 min, followed by 35 cycles at 95 °C for 30 sec, and 61 °C for 30 sec and 72 °C for 35 sec. To normalize the expression of RNAs, β-actin was used and the relative changes in mRNAs were calculated by the method of 2^−ΔCt^. The sequences of all primers are listed in Table 1(Tab. 1).

### Western Blot analysis

The treated cells were washed and resuspended in RIPA buffer (25 mM Tris-HCl (pH 7.4), 150 mM NaCl, 2 mM EDTA, 1.0 % Triton X-100, 1.0 % sodium deoxycholate, and 0.1 % SDS) containing protease and phosphatase inhibitors, on ice for 30 min. The lysates were then centrifuged at 14,000×g, 4 °C for 15 min. The supernatants were collected and the protein concentration was measured using the Bradford method. An equal amount (50 µg) of protein in each sample was boiled in a loading buffer for 5 min and loaded into each lane of 10 % SDS-PAGE gel and the polypeptides were electrotransferred to a PVDF. The membrane was blocked with 5 % skim milk in TBST (150 mM NaCl, 10 mM Tris (pH 8.0), and 0.05 % Tween-20) at 4 °C for 2 hrs and were then incubated with BVR-A and β-actin primary antibodies at a dilution of 1:600 and 1:1000 at 4 °C overnight. Following washing with TBST solution, three times, it was incubated with anti-rabbit or anti-mouse HRP conjugated secondary antibody at a dilution of 1:3000 for 2 hrs at room temperature and washed in TBST again. To detect the antigen-antibody complexes, ECL chemiluminescence reagents were used, and to measure western blot bands, Image Lab 4.1 software (National Institutes of Health, USA) was used.

### Statistical analysis

Data analysis was performed using GraphPad Prism 6.0 software. One-way analysis of variance (ANOVA) followed by Dunnett's post hoc test was used. Mean ± standard deviation (SD) of at least three independent experiments was presented. P ≤ 0.05 was considered statistically significant.

## Results

### Molecular docking

**Figure 1:**
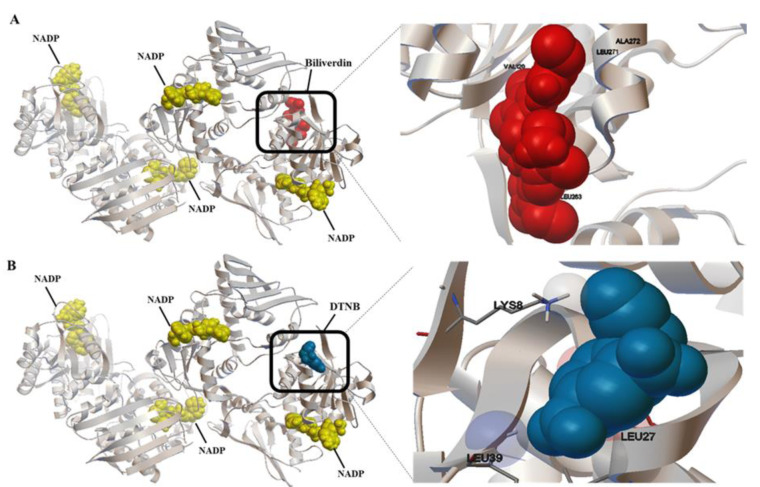
Figure 1: Three-dimensional conformation and close-up view of the BVR-A binding site with biliverdin (A), and DTNB (B).

**Figure 2:**
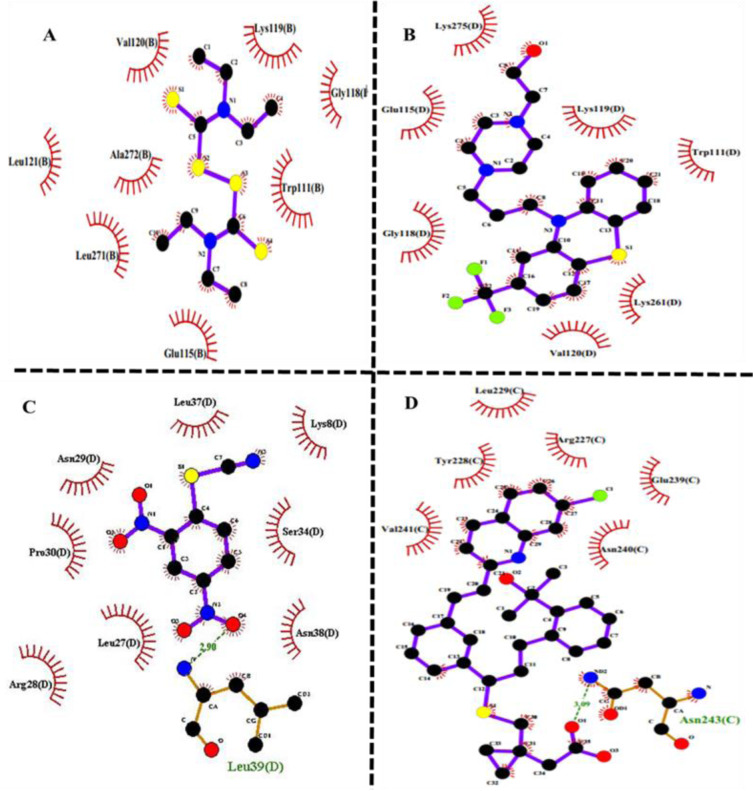
Two-dimensional scheme of interactions of BVR-A with the docked molecule: A) Disulfiram, B) Fluphenazine dihydrochloride, C) DTNB, and D) Montelukast, obtained by Ligplot+ software. Receptor residues involved in hydrophobic interactions are represented by black semicircular arcs, and hydrogen bonding shows in green dotted lines. Carbon, oxygen, nitrogen, and fluorine atoms are displayed in filled black, red, blue, and green circles, respectively.

To determine the active site of BVR-A, it was first docked with its substrate, biliverdin. The coordinates of the grid box utilized for docking are illustrated in Table 2(Tab. 2). Also, the interacting amino acid residues of BVR-A with biliverdin are shown in Figure 1(Fig. 1). Compounds used as ligands in the pharmacophore model are presented in Table 3(Tab. 3). Four compounds (Disulfiram, Fluphenazine dihydrochloride, Montelukast, and DTNB) were selected for further molecular docking. The results obtained from the molecular docking of these compounds with BVR-A are shown in Table 4(Tab. 4). The binding of free energy (ΔGbind) is calculated by the determination of binding strength between these ligands and BVR-A (Table 4(Tab. 4)). These results indicate that the binding energy among tested compounds is different and the lowest binding energy is related to the DTNB. The binding energy between DTNB and BVR-A is -2.84 Kcal/mol which shows a greater affinity for BVR-A than the other ligands (Table 4(Tab. 4)). Ligplot^+^ software, as a two-dimensional schematic, represents the interactions between ligands and residues within the binding site of BVR-A (Figure 2(Fig. 2)). The result of MD showed that DTNB induces a conformational change in BVR-A structure which inhibits binding of BVR-A to biliverdin.

### Biliverdin decreases cell viability

**Figure 3:**
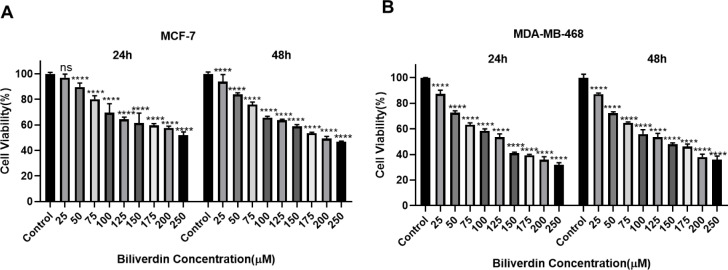
Figure 3: Induction of cell death by exogenous biliverdin in breast cancer cell lines. MCF-7 (A) and MDA-MB-468 (B) cells were treated with different concentrations of biliverdin (from 25 to 250 µM) for 24 and 48 hrs. The cell viability was evaluated by MTT assay. The data are presented as mean ± SD (three separate experiments). **P<0.01, ***P<0.001, and ****P<0.0001, denote means significantly different from control cells.

As shown in Figure 3A and B(Fig. 3), biliverdin inhibited the viability of MCF-7 and MDA-MB-468 in a time- and dose-dependent manner. The cell death of MDA-MB-468 for 250 μM of biliverdin was more than MCF-7 cells (P<0.0001). The effective dose of biliverdin for the inhibition of 50 % of cell growth (IC_50_), after 24 hrs of treatment, was 247.4 µM for MCF-7 and 168.9 μM for MDA-MB-468. In the subsequent experiments, 250 µM and 170 µM of biliverdin have been used as optimum concentrations for MCF-7 and MDA-MB-468 respectively.

### Biliverdin induces cell apoptosis 

**Figure 4:**
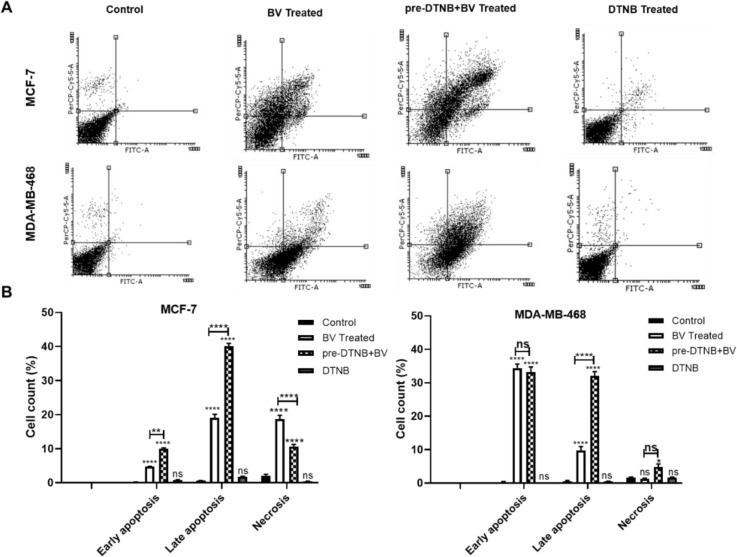
Induction of different modes of cell death by biliverdin, biliverdin plus DTNB, and DTNB in MCF-7 and MDA-MB-468 cell lines. (A) MCF-7 and MDA-MB-468 cell line treated with biliverdin for 24 hrs. DTNB was pretreated for 12hrs prior to biliverdin treatment. After 24 hrs of incubation with biliverdin, the percentage of cell death (early and late apoptosis) was significantly increased in both cell lines. Necrosis was also increased in these cell lines, although not significantly in MDA-MB-468 cells. Pretreatment with DTNB has significantly decreased cell death after biliverdin treatment. (B) Quantification of Figure A. Data is represented as mean ± SD (three separate experiments). *P<0.05, **P < 0.01, ***P < 0.001, ****P < 0.0001 denotes a mean significantly different from control cells, ns not significantly different from control cells (BV= biliverdin)

To elucidate whether the effects of biliverdin on the MCF-7 and MDA-MB-468 cells were associated with the induction of apoptosis, annexin V‐ PI staining was used. The percentages of early, late apoptotic, and necrotic cells after incubation of both cells with biliverdin are shown in Figure 4A and B(Fig. 4). Biliverdin treated MCF-7 cells showed a significant increase in the early (4.80 %) and late (18.32 %) apoptosis compared to controls (Figure 4A and B(Fig. 4)) (P<0.0001). As shown in Figure 4A and B(Fig. 4), MDA-MB-468 treated cells with biliverdin showed significant cell apoptosis (33.54 % early and 8.04 % late apoptosis) (P<0.0001). However, there were no significant differences in the necrosis (P =0.96). Taken together, these findings indicate that biliverdin induces apoptosis in breast cancer cells.

The effect of DTNB, as a BVR-A inhibitor, on the biliverdin-induced cell apoptosis was examined. As shown in Figure 4A and B(Fig. 4), MCF-7 treated cells with biliverdin plus DTNB exhibited a significant apoptotic effect when compared to those treated with biliverdin alone (9.83 % early and 40.93 % late apoptosis) (P < 0.01, P<0.0001). Also, treatment of MDA-MB-468 cells with biliverdin plus DTNB increased cell apoptosis when compared to those treated with biliverdin alone (30.85 % late apoptosis) (P<0.0001). DTNB did not affect cell viability (Figure 4A and B(Fig. 4)).

### Biliverdin induces intrinsic apoptotic pathways 

**Figure 5:**
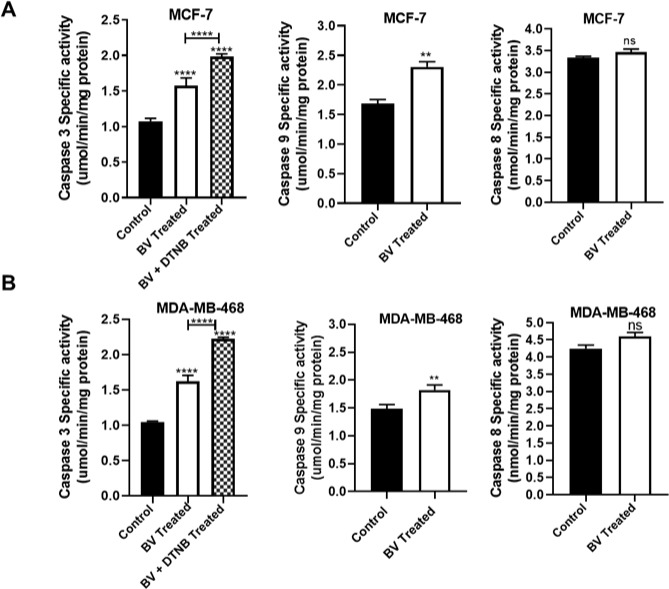
Specific activities of caspases in the cell lysate of breast cancer cell lines, MCF-7 and MDA-MB-468 cells, treated with biliverdin for 24 hrs. DTNB was pretreated for 12 hrs prior to biliverdin treatment after incubation of MCF-7 (A) and MDA-MB-468 (B) cells with Biliverdin, the activity of caspase-3, -9, and -8 were increased significantly, using an enzymatic assay. In contrast, biliverdin did not affect the activation of caspase‐8. Pretreatment with DTNB significantly increased the activity of caspase-3 after biliverdin treatment. The results were presented as the mean values ± SD. **P < 0.01, ***P < 0.001 denotes a mean significantly different from control cells.

As presented in Figure 5A and B(Fig. 5), the activity of caspase-3 was significantly increased after treatment of MCF-7 and MDA-MB-468 cells with biliverdin (1.46 and 1.54 times more than control, respectively) (P < 0.0001). Furthermore, caspase-3 activity was significantly increased after treatment of MCF-7 and MDA-MB-468 cells with biliverdin plus DTNB (1.98 and 2.22 times more than control, respectively) (P < 0.0001). 

To distinguish whether biliverdin initiates apoptosis via the intrinsic or extrinsic pathway, the activities of caspase‐8 and ‐9 were determined. Following treatment of MCF-7 and MDA-MB-468 cells with biliverdin, the activity of caspase-9 has been increased significantly (1.36 and 1.22 times more than control) (P < 0.01, P < 0.01) (Figure 5A and B(Fig. 5)). In contrast, biliverdin had no significant effect on the activity of caspase-8 (Figure 5A, and B(Fig. 5)).

### Biliverdin induces BVR-A and AhR expression 

**Figure 6:**
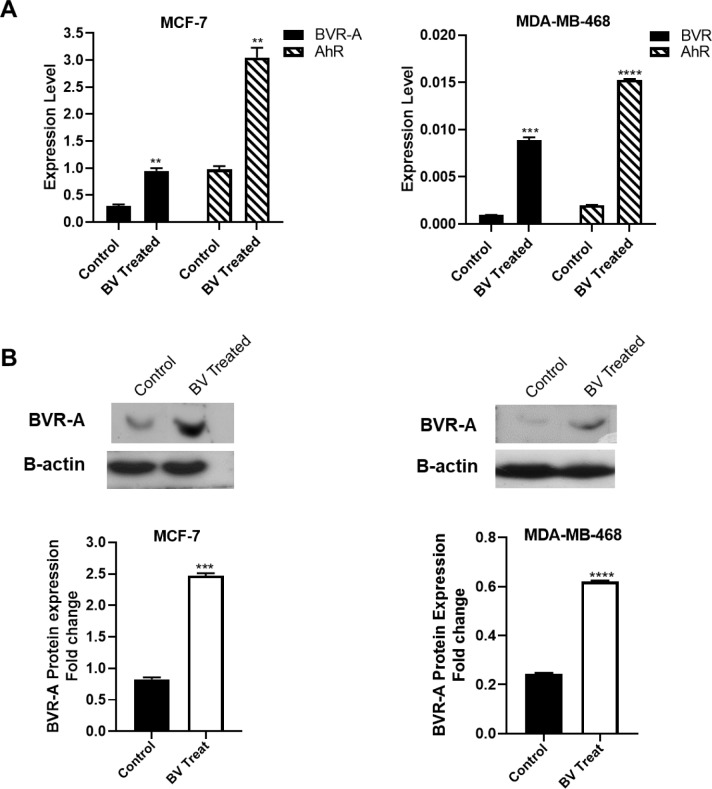
mRNA level of BVR-A and AhR (A) and protein level of BVR-A (B) in the biliverdin-treated breast cancer cell lines. β-actin was used for normalization in real-time-PCR analysis and western blot. *P<0.05, **P < 0.01, ***P < 0.001, and ****P < 0.0001 denotes a mean significantly different from control cells.

BVR-A gene expression and protein levels were assessed in both breast cancer cell lines after treatment with biliverdin for 24 hrs. As shown in Figure 6A(Fig. 6), the expression level of the BVR-A gene was up-regulated in MCF-7 and MDA-MB-468 following treatment with biliverdin (P<0.01, P<0.001). A significant increase was observed in the BVR-A protein level after the treatment of both cell lines with biliverdin (P<0.0001) (Figure 6B(Fig. 6)). The gene expression and protein levels of BVR-A were higher in MCF-7 than those of MDA-MB-468 cells. The expression level of AhR gene was also up-regulated in the biliverdin treated MCF-7 and MDA-MB-468 cells (P<0.01, P<0.0001) (Figure 6A(Fig. 6)).

### Determination of intra- and extracellular bilirubin 

**Figure 7:**
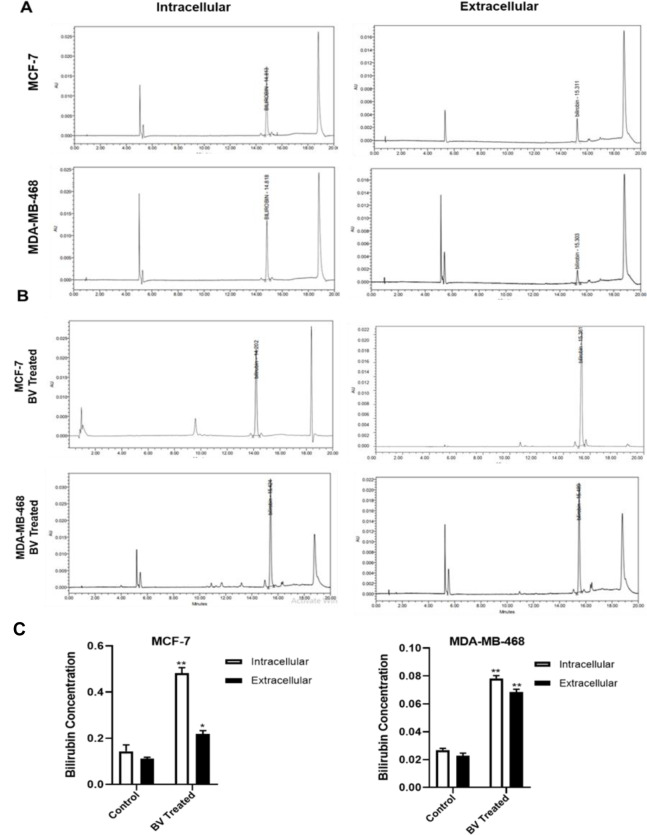
HPLC chromatograms of bilirubin in intra- and extracellular of in control cells (A) and treated cells (B). Detection wavelength of 453 nm. (C) Quantification of intra- and extracellular levels of bilirubin. The data are expressed as the mean ± SD, *P<0.05, **P < 0.01, ***P < 0.001, and ****P < 0.0001 denotes a mean significantly different from control cells.

It has been demonstrated that intra- and extracellular concentrations of bilirubin in MCF-7 cells were higher than those of MDA-MB-468 cells (Figure 7A and C(Fig. 7)). Treatment of MCF-7 cells with biliverdin for 24 hrs showed that intra- and extracellular bilirubin levels were 2.60 and 2.10 times higher than those of untreated cells (P < 0.01, P<0.05) (Figure 7B and C(Fig. 7)). In the treated MDA-MB-468 cells with biliverdin, the bilirubin concentration was significantly increased in both intra- and extracellular fluids (2.96 and 3.27 times higher than control) (P < 0.01, P < 0.01). 

### Measurement of BVR-A activity

**Figure 8:**
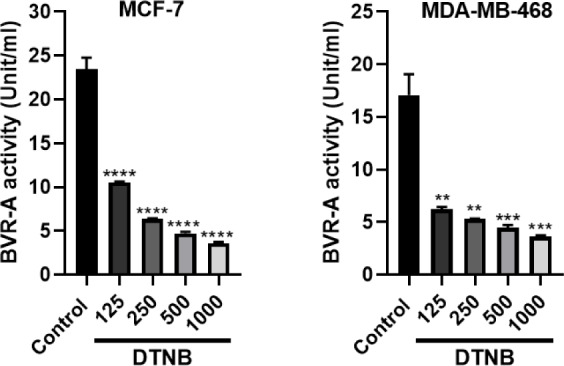
Inhibition of BVR-A by DTNB in breast cancer cell lines. MCF-7 and MDA-MB-468 cells were treated with different concentrations of DTNB (from 125 to 1000 µM) for 12 hrs. The data are presented as mean ± SD (three separate experiments). **P<0.01, ***P<0.001 and ****P<0.0001, denote means significantly different from control cells.

Before and after the treatment of the cells with DTNB, the BVR-A activity was evaluated. MCF-7 and MDA-MB-468 were incubated with different concentrations of DTNB for 12 hrs. IC50 was 59.06 for MCF-7 and 40.25 μM for MDA-MB-468 at 12 hrs. The results showed that BVR-A activity was significantly reduced, which suggests DTNB is a potent inhibitor (Figure 8(Fig. 8)). 

## Discussion

In the present study, it has been found that biliverdin, in a dose-dependent manner, reduces the cell viability and induces cell apoptosis in both breast cancer cell lines, MCF-7 and MDA-MB-468. In agreement, other investigations have shown that biliverdin inhibited the growth of various cell lines. It has been reported that treatment of head and neck cells with biliverdin directed the cells to apoptosis (Zheng et al., 2014[34]). In rat and mouse VSMCs, it has been demonstrated that biliverdin did not persuade apoptosis although it suppressed the cell-cycle progression at the G0/G1 phase (Ollinger et al., 2007[20]). However, pretreatment of renal cells by biliverdin increased the cell resistance to apoptosis that was induced by cisplatin via inhibition of oxidative stress (Lv et al., 2016[17]). Different effects of biliverdin on apoptosis have been reported, which might be dependent on the various concentrations of biliverdin that were used in various experiments. It has been reported that biliverdin, at millimolar concentrations, induced cell apoptosis (Ollinger et al., 2007[19]), whereas at micromolar levels it inhibited apoptotic response (Busserolles et al., 2006[6]). 

In this study, it has been demonstrated that biliverdin induced caspase-3 dependent apoptosis in breast cancer cell lines. Induction of caspase-dependent apoptosis by biliverdin has also been reported in other malignant cells (Rodrigues et al., 2002[25]; Keshavan et al., 2004[13]). Caspase-3 is the most important executor in both intrinsic and extrinsic pathways (Jiang et al., 2020[12]). It is activated by caspase-8 (in the extrinsic pathway of apoptosis) and caspase-9 (in the intrinsic pathway of apoptosis) (Jiang et al., 2020[12]). In the present study, it has been further shown that apoptosis induced with biliverdin is mediated by an intrinsic pathway, as evidenced by activation of caspase‐9. However, Keshavan et al. failed to demonstrate the effects of biliverdin on caspases (Keshavan et al., 2004[13]). 

Regarding the relationship between biliverdin and BVR, and to exhibit that these effects are induced by either biliverdin or bilirubin, the conversion of biliverdin to bilirubin was blocked using DTNB, a BVR-A inhibitor. Considering the BVR-A inhibition, it has been demonstrated that DTNB, with a lower binding energy score (-2.84 Kcal/mol), possesses the highest binding affinity for BVR-A than the other studied ligands (Table 4(Tab. 4)). The results demonstrated that cell death induced by biliverdin plus BVR-A inhibitor was higher than that induced by biliverdin alone. Using several complementary procedures including annexin V-PI staining and assessment of caspase‐3 activity, it has been confirmed that in the presence of DTNB, induction of apoptosis was higher than that of biliverdin alone. There are three possible mechanisms to describe these effects on the cells. Firstly, reduction in the basal level of endogenous bilirubin which is a potent antioxidant (Bianco et al., 2020[3]), and secondly elevation in the level of biliverdin which has a negative feedback inhibition on the activity of heme oxygenase1 (HO-1) (Salim et al., 2001[26]). The third mechanism is the inhibitory effect of biliverdin on NFκB activation, which has been associated with apoptotic response (Gibbs and Maines, 2007[9]). It has also been reported that the inhibitory effect of biliverdin on cell proliferation and angiogenesis relies on its antioxidant activity and is independent of its conversion to BR by BVR-A (Zheng et al., 2014[34]).

The expression of BVR-A is tissue-specific since it is expressed in the lung (Liu et al., 2017[15]) and liver cancers, but not in ovarian cancer, and its expression was up-regulated in skin cancer cells (Arena et al., 2015[1]; Zhang et al., 2016[32]). In the current study, the results revealed that mRNA and protein levels of BVR-A were higher in MCF-7 than those of MDA-MB-468. Since the measurement of intra- and extracellular levels of bilirubin could provide a deep understanding of the mechanisms, bilirubin was measured using the HPLC method. The present results revealed that intra- and extracellular concentrations of bilirubin were higher in MCF-7 than those of MDA-MB-468 cells because the expression of BVR-A was also higher in MCF-7. The intracellular concentration of bilirubin could be influenced by cellular uptake, mitochondrial oxidative enzymes, as well as a different pattern of BVR-A expression in the cells and tissues (Bianco et al., 2020[3]). The results showed that bilirubin was significantly higher in the biliverdin treated cells, suggesting that biliverdin, in part, is converted to bilirubin in breast cancer cells. 

Interestingly, an increase in AhR gene expression was observed in biliverdin-treated breast cancer cells. AhR is the only binding site for biliverdin (Gazzin et al., 2016[8]). It has a potential role in the regulation of cell proliferation, cell-cycle distribution, and cell apoptosis (Puga et al., 2009[24]). It has been shown that activation of AhR resulted in cell proliferation, while its stimulation by endogenous substrates may induce cell cycle arrest, as shown in LoVo human colon cancer cells (Yin et al., 2016[31]). These results suggest that activation of AhR by biliverdin may contribute to the direction of the cells to the apoptosis (Phelan et al., 1998[23]).

## Conclusion

In the present study, it has been found that in breast cancer cells, biliverdin induces cell apoptosis through intrinsic pathways, as evidenced by caspase‐3 and -9 activities. In addition, treatment of the cells with biliverdin plus DTNB elevated cell apoptosis. Incubation of both cell lines with biliverdin increased the intra- and extracellular level of bilirubin, i.e., biliverdin is converted to bilirubin. Since BVR-A was highly expressed in MCF-7, compared to that of MDA-MB-468, bilirubin was also higher in this cell line. Taken together, it has been shown that the effect of biliverdin is complex and needs further investigation. 

## Declaration of competing interest

The authors declare that there are no conflicts of interest regarding the publication of this paper.

## Figures and Tables

**Table 1:**
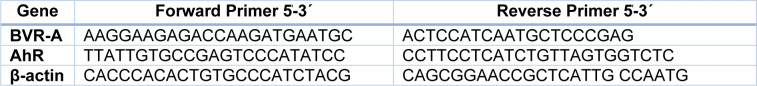
Primer sequences for qRT-PCR analysis

**Table 2:**
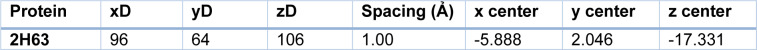
Grid box coordinates for BVR-A

**Table 3:**
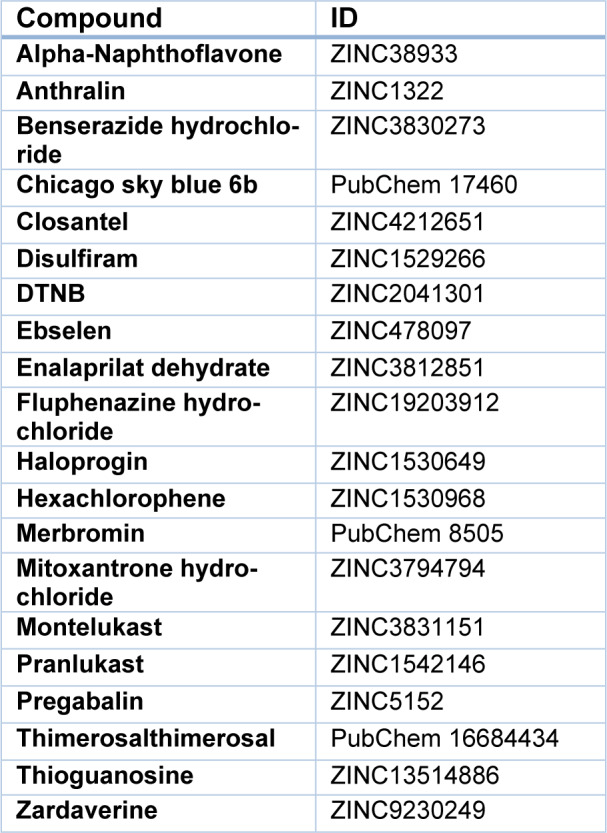
Compounds were used as ligands in the pharmacophore model.

**Table 4:**
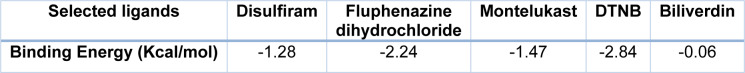
The binding energy of selected ligands against the BVR-A
